# Managing sleep apnea: long-term outcomes from a comprehensive, patient-centered treatment care pathway

**DOI:** 10.3389/frsle.2025.1593874

**Published:** 2025-06-10

**Authors:** Heidi Doss Riney, Frances P. Thorndike, Jon S. Agustsson, Snorri Helgason, Karina Hauser, Alp Sinan Baran, Thomas Kauss, Gregory David Salinas, Samantha Edington, Emerson M. Wickwire

**Affiliations:** ^1^Nox Health, Alpharetta, GA, United States; ^2^CE Outcomes, LLC, Brimingham, AL, United States; ^3^Division of Pulmonary, Critical Care, and Sleep Medicine, Department of Medicine, University of Maryland School of Medicine, Baltimore, MD, United States

**Keywords:** sleep apnea, positive airway pressure (PAP), adoption, adherence, persistence, quality of care (QoC), comprehensive care

## Abstract

**Introduction:**

Obstructive Sleep Apnea (OSA) is a chronic disease requiring life-long care, with clear benefit for those who remain adherent to positive airway pressure (PAP) therapy. Despite the efficacy of PAP, adherence to treatment has historically been low. The purpose of this study was to determine the impact of a streamlined OSA care pathway on quality of care and PAP adoption/adherence.

**Methods:**

Two retrospective cohort studies were performed based on real-world data gathered as part of routine clinical care within a large comprehensive sleep care program. In Study 1, quality of OSA care was assessed by evaluating days spent between treatment steps of the care pathway, including time to diagnosis and treatment initiation. In Study 2, long-term PAP adoption, adherence, and persistence data were analyzed; PAP average minutes used per night and average nights per week were also calculated.

**Results:**

In Study 1, patients (*n* = 42,687) typically underwent telehealth consultation within 5 days of OSA screening; completed Home Sleep Apnea Testing (HSAT) within 12 days from physician consultation; received testing results and recommendations within 9 days of completing HSAT; and initiated PAP within 8 days of a diagnosis. In Study 2 (*N* = 4,907), 84.3% of patients placed on therapy adopted therapy and 80.6% of those who adopted demonstrated short-term adherence. 82.6% of patients demonstrated long-term PAP adherence 1 year after adoption and 74.2% of patients persisted with PAP 2 years after adoption. PAP usage rates increased over time. By year 3, patients (*n* = 3,067) used their PAP device an average of 6.0 days per week, with mean usage of 6.4 h per night.

**Discussion:**

Length of time between treatment steps was shorter than published reports. Rates of both short- and long-term adherence and persistence to PAP therapy were also higher than those observed in most prior studies. Average nightly use and nights per week used trend upward across the 3 years. These findings suggest that a comprehensive OSA care approach can effectively help more patients get on therapy and stay on therapy, providing an opportunity for the health and economic benefits reported in the literature.

## Introduction

Obstructive sleep apnea (OSA) is a common and costly medical condition that impacts ~936 million individuals worldwide (Benjafield et al., 2019). The condition is characterized by partial or complete airway collapse during sleep, resulting in intermittent airflow reduction and breathing pauses throughout sleep, each lasting 10 or more seconds and resulting in blood oxygen desaturation, sympathetic arousals, and sleep fragmentation. OSA is a chronic disease requiring life-long care, with clear benefit for those who stay on recommended treatment. In contrast, untreated OSA creates a significant burden from all perspectives; this burden is for patients through worse health outcomes and diminished quality of life, for employers through decreased productivity and increased accident risk, and for payers through higher healthcare and disability costs. Untreated OSA increases risk for cardiovascular disease, depression, diabetes, and premature death, to name several consequences (Garbarino et al., 2020; Gottesman et al., 2024; Ogilvie and Patel, 2018; Yeghiazarians et al., 2021).

A growing body of literature highlights the economic benefit of OSA care. For example, in 2016, the American Academy of Sleep Medicine commissioned a paper that reported total OSA-related costs of untreated OSA of $150 billion per year with potential cost savings from OSA care of $100 billion per year, as measured in USD in 2015 (Watson, 2016; Frost and Sullivan, 2016). A 2019 systematic review examined the impact of OSA on monetized economic outcomes and found that 15 of 18 studies resulted in positive economic benefit (Wickwire et al., 2019). More recently, a white paper reported findings from an administrative claims analysis showing that successful OSA care was associated with ~$5,400 reduction per patient in total healthcare costs over 2 years (Risk Strategies Consulting, 2024).

The first-line treatment for OSA is positive airway pressure (PAP) therapy. When used as prescribed (i.e., all night every night), PAP effectively eliminates OSA. Unfortunately, claims analyses demonstrate that, as seen in other chronic disease conditions, adoption and adherence to PAP is suboptimal, with up to 51% of patients declining to try PAP at all, 25% of patients maintaining adherence for 1 year, and only 11% persisting to treatment at 2 years (Risk Strategies Consulting, 2024). A commentary on the challenges with adherence noted that 46–83% of patients with obstructive sleep apnea have been reported to be nonadherent to treatment, when adherence is defined as at least 4 h of nightly use (Weaver and Grunstein, 2008). In traditional care models, one barrier to PAP is a demanding patient journey that typically includes primary care, sleep specialty care, diagnostic sleep testing, coordination with durable medical equipment (DME) providers, troubleshooting, and the need to coordinate ongoing support (Wickwire et al., 2024). Even when completed successfully, this OSA patient journey often takes many months and significant effort from patients.

The purpose of this study was to determine the potential impact of a streamlined OSA care pathway on quality of care as well as PAP adoption and adherence. We report findings from two retrospective analyses of clinical data from a national sleep telemedicine provider in the US to determine whether an optimized OSA pathway can result in better outcomes than those reported in the literature. From a research perspective, all patients would ideally be followed over time, from OSA screening through long-term outcomes. In a real-world setting, however, such optimal study designs are often not possible. For example, the OSA care model described herein has been refined and continues to improve over time. As a result, not all relevant metrics (e.g., time between treatment steps) are available for all patients. At the same time, examination of long-term outcomes is vital. Given this, our approach is to provide results from two non-overlapping cohorts that span nearly a decade of real-world clinical care. Study 1 includes relatively recent data (2021–2023) and aims to provide insight into a refined and continually improving OSA care model. Study 2 includes slightly older, non-overlapping data (2016–2020) and aims to provide insight into long-term adherence and other outcomes once patients are prescribed treatment. By including two cohorts, this paper aims to provide a comprehensive overview of a real-world OSA care program over time and to enable practical comparisons against more traditional, often disjointed OSA care models.

## Methods

### Study design and overview

This paper presents results of two related retrospective cohort studies of real-world data gathered as part of routine clinical care for patients treated within a large sleep telemedicine program in the U.S. In Study 1, we examined the quality of OSA care, including time to diagnosis, time between positive diagnosis and treatment adoption, as well as median number of days between multiple treatment steps of the care pathway. Study 2 presents long-term PAP adoption, adherence, and persistence data of a cohort of OSA patients for whom longer-term follow-up data were available (>3 years), as well as average PAP usage data in nights per week used and minutes per night used.

### Description of OSA care pathway

**Figure 1:**
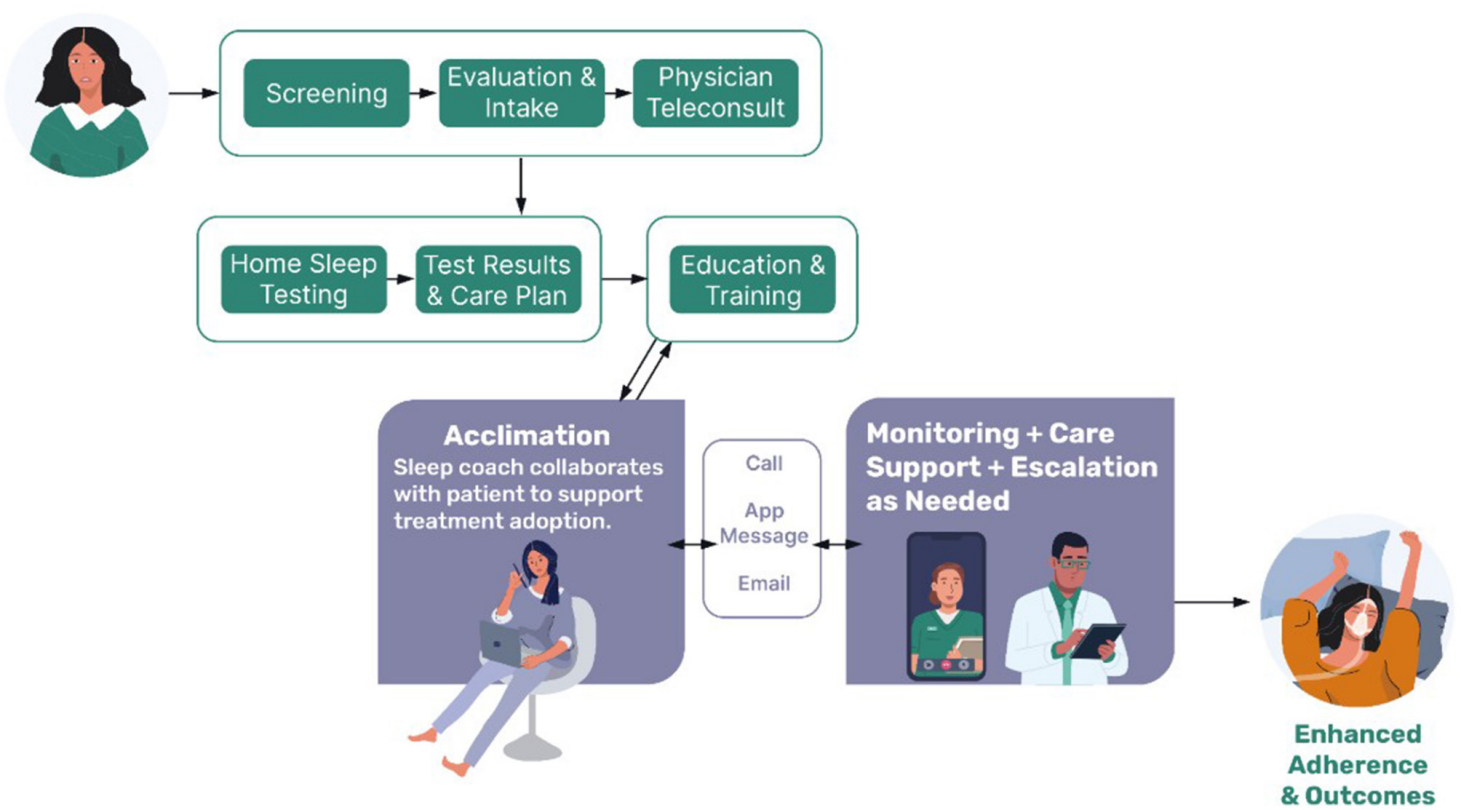
Schematic of the comprehensive sleep care pathway. Potential patients complete an online screening questionnaire and validated patient reported outcome measures. This detailed sleep and health information helps to guide a telehealth consultation with a board-certified sleep physician to clinically assess for sleep disorders, including sleep apnea. If indicated, a home sleep apnea test is conducted. Results are discussed with the patient, and a care plan is established. If patients opt into PAP therapy, they receive additional education and training on the PAP device. Support and monitoring are provided by a behavioral care team to help acclimate the patient and troubleshoot any initial difficulties. Once acclimated, patients are supported through device monitoring and are contacted by clinical staff as indicated. The clinically integrated team includes board-certified sleep physicians, a nurse practitioner, medical support team, respiratory therapists, and a behavioral care team.

Figure 1 depicts the OSA care model and text below describes the treatment steps. Patients follow a clearly defined sequence of care, supported throughout by a clinically integrated care team consisting of a board-certified sleep physician, nurse practitioner, respiratory therapist, and behavioral care manager, moving the patient from initial screening (Screening) through completion of patient reported history and patient reported outcomes (Evaluation and Intake) and on to the physician teleconsult (Teleconsult). If the physician suspects sleep apnea, home sleep apnea testing (HSAT) is ordered and sent to the patient. Contraindications to HSAT were taken into consideration in accordance with AASM practice guidelines. If a patient did not qualify for home testing, the clinical team arranged local in-lab PSG testing.

Upon completion of the HSAT, testing results are interpreted by the physician, followed by discussion of test results and recommended treatment plan (Test Results and Care Plan). If patients opt into PAP treatment, they receive a device from the care manager (including both the PAP machine and consumables as needed from a variety of companies), as well as more education about OSA, their prescribed treatment, and preparing for adoption/acclimation (Education and Training). Patients begin with autotitrating PAP but depending on their clinical needs, the mode can be switched to fixed-pressure PAP, or the device can be changed to bilevel-PAP or adaptive servo-ventilation.

Interaction with patients is made on both a proactive and reactive basis. Sleep Coaching is conducted by behavioral care managers and medical managers throughout the patient's entire 1-year treatment cycle (which can then be renewed for additional years). During Acclimation (the first 30–45 days of treatment) the care team works with the patient to engage in therapy successfully, using a variety of methods of communication (text, email, phone). Contact occurs, on average, every 5 days. After acclimation, the care team shifts to a supportive role to monitor and promote long-term adherence and persistence to therapy (Monitoring + Care Support). For example, the care team monitors usage parameters, and the PAP machine-calculated residual estimated apnea-hypopnea index (rAHI), contacting the patient when intervention is needed. Escalations to the medical team occur when warranted. If compliance remains above 70%, patient contact occurs on average every 45–90 days. If compliance dips below 70%, contact is made every 7 days until compliance rises back above 70%. The care team also handles communication with the patient around DME resupply, behavioral, technical, and medical barriers. Patients also reach out on their own if they have questions or concerns between those planned touchpoints. Further description of the care pathway has been recently published (Salinas et al., 2025).

### Home sleep apnea testing

In both Study 1 and Study 2, HSAT was performed using Nox Medical T3 and T3s™ devices, which include channels to monitor airflow, snoring, respiratory effort, pulse oximetry, and body position. When additional testing was necessary to further evaluate patients with inconclusive HSAT results, electroencephalogram (EEG) and electrooculogram (EOG) channels were included. Scoring was performed using the AASM Manual for the Scoring of Sleep and Associated Events; hypopneas were scored using the 1A (for HSAT) or 2A (for HSAT + EEG/EOG) hypopnea scoring rules. As per AASM guidelines, severity of sleep apnea was graded based on the respiratory event index (REI) for HSAT or apnea-hypopnea index (AHI) for HSAT + EEG/EOG, including mild (5–15 events per h), moderate (15–30/h), and severe (>30/h) (American Academy of Sleep Medicine, 2014).

### Study 1: quality of OSA care

The participants of this portion included employees at US-based companies who underwent OSA screening between 2021 and 2023 as part of a contracted OSA care management program. Broadly speaking, employees with possible OSA were identified via multiple pathways, including provider referral (e.g., from onsite health clinics), self-referral (e.g., based on workplace education programs), or regulatory necessity (e.g., in the trucking industry). Inclusion criteria were completion of OSA screening and insurance coverage for OSA care. To maximize generalizability of findings, no specific exclusion criteria were applied. Dependents (e.g., family members) were also eligible to participate.

All participants completed OSA screening. Demographic and clinical characteristics were assessed via online questionnaire and/or telehealth video consultation with a board-certified sleep physician. Geographical location was categorized as rural/urban based on the 2010 USDA Economic Research Service (ERS) census definitions (USDA Economic Research Service, 2012). Data were captured within a proprietary electronic health record system used for OSA care management. Quality of OSA care was defined as time in days between treatment steps, including time from screening to meeting with a physician, time from meeting with the physician to implementing Home Sleep Apnea Testing (HSAT), and time from diagnosis and treatment recommendation to treatment initiation. For time to event analyses, participant completion of the initial OSA screening was considered Day 0, and time was measured either in days from this date or days between identified steps in the treatment pathway.

Descriptive statistics were used to characterize participants, including means with standard deviation (SD), medians with interquartile ranges (IQR), and counts or proportions for categorical variables.

### Study 2: long-term PAP adoption, adherence, and persistence

PAP data was obtained via cloud-connected devices. Patients without internet access mailed back their SD memory cards, and adherence data were manually uploaded into the clinical database. Descriptive statistics were used to characterize participants, with means and SD for continuous variables, and counts and proportion for categorical variables. To present a comprehensive view of usage, we evaluated several variables that together can form a composite view. Adoption of PAP therapy was defined as using PAP for >60 min in a single day, reflecting an attempt to use PAP (and not simply verify machine functionality). Ninety-day adherence to PAP therapy was defined as meeting the CMS adherence threshold of >4 h/night on >70% of nights within a 30-day period, with symptomatic benefit, within the first 90 days. Long-term adherence (1 year) and persistence (2 or more years) to PAP therapy was characterized by patient benefit from using PAP, demonstration of consistent effort using their PAP device, and a desire to continue using PAP in the following year (Year 1 adherence). When these criteria are met, they move into a subsequent year of treatment. If they meet these criteria for 2 years, they move into a third year (and so on). Details of usage within a given year are then further clarified by mean number of days per week and hours per night of use at 1-, 2-, and 3-year follow-up. Finally, effectiveness of PAP therapy was evaluated at each point based on PAP machine-calculated rAHI.

## Results

### Study 1: quality of OSA care

The final sample for Study 1 included *N* = 42,687 individuals screened for OSA between 2021 and 2023. Table 1 presents the demographic baseline characteristics. Forty-seven percent of participants self-identified as female (47.0%). Most participants (59.5%) were between the ages of 36–55 years. Two-thirds of the sample lived in rural ZIP codes. Table 2 summarizes OSA quality of care metrics, including the time in days between multiple treatment steps. Median values show that patients typically underwent telehealth consultation within 5 days of OSA screening and completed HSAT within 12 days from physician consultation. Median values also show that testing results were typically reviewed with patients within 9 days of completing HSAT. Lastly, patients typically initiated PAP therapy within 8 days of receiving their diagnosis and recommended treatment plan.

**Table 1:** Study 1 demographics of treatment pathway analysis.

**Demographic**	**Total (*N* = 42,687)**
**Gender**	
% Female	47.0%
% Male	46.1%
% Not identified	6.9%
**Age**	
18–25	1.3%
26–35	16.7%
36–45	27.9%
46–55	31.6%
56–65	19.2%
>65	3.2%
**Geographic location**	
Rural	67.0%
Urban	32.8%
Unknown	0.2%

**Table 2:** Summary of OSA quality of care metrics.

**Description of timeframe**	**Time in Days: median (IQR [25%-75%])**
Days elapsed from OSA screening to completion of teleconsultation with a physician	5 [3–12]
Days elapsed from telehealth consultation with physician to implementation of Home Sleep Apnea Testing	12 [9–16]
Days elapsed from HSAT completion to review of HSAT results, diagnosis, and treatment recommendations	9 [7–12]
Days elapsed from positive OSA diagnosis to PAP initiation	8 [5–14]

Days elapsed between steps in the OSA care pathway begins with a sample of 42,687.

OSA, obstructive sleep apnea; HSAT, home sleep apnea testing; PAP, treatment with positive airway pressure; IQR, Interquartile range.

### Study 2: long-term PAP adoption, adherence, and persistence, and usage

Four thousand nine hundred and seven patients who tested positive for sleep apnea between January 2016 and December 2020 were included (Table 3) to observe long-term PAP adoption, adherence, and persistence. Patients were predominantly male (82.4%). The most common age ranges were 46–55 (31.1%) and 56–65 (33.7%). Patients were more likely to be from urban (87.3%) rather than rural (12.6%) environments. In this patient group, 47.2% had a baseline REI/AHI of 30 or more events per hour, as measured by the standard Type III HSAT or with added EEG/EOG, indicating severe sleep apnea.

**Table 3:** Study 2 demographics of cohort for adoption analysis.

**Demographic**	**Total (*N* = 4,907)**
**Gender**	
% Female	17.3%
% Male	82.4%
% Not identified	0.2%
**Age**	
18-24	0.04%
26-35	4.9%
36-45	19.2%
46-55	31.1%
56-65	33.7%
>65	11.2%
**Geographic location**	
Rural	12.6%
Urban	87.3%
Unknown	0.1%
**Severity of sleep apnea**	
Mild	23.6%
Moderate	29.2%
Severe	47.2%

This cohort included 4,907 patients diagnosed with OSA, of whom 4,134 (84.3%) adopted PAP therapy (Table 4). Of the 4,134 who adopted PAP, 3,332 (80.6%) demonstrated short-term 90-day adherence, meeting CMS adherence criteria during the first 90 days. In terms of long-term adherence, 3,414 (82.6%) patients demonstrated adherence to PAP therapy at >1 year, and 3,067 (74.2%) patients persisted with PAP therapy still showing adherence at >2 years.

**Table 4:** Use of PAP therapy across multiple years.

**Adoption, adherence, and persistence to PAP therapy**	
Adoption^*^	84.3% (4,134/4,907)
90-day Adherence^**^	80.6% (3,332/4,134)
1-year Adherence^†^	82.6% (3,414/4,134)
2-year Persistence^‡^	74.2% (3,067/4,134)
**Mean days of PAP use per week** ^§^	
Year 1 (n = 3,905)	5.49 (SD 2.11) days/week
Year 2 (n = 3,414)	5.76 (SD 1.97) days/week
Year 3 (n = 3,067)	6.00 (SD 1.73) days/week
**Mean hours of PAP use per night** ^§^	
Year 1 (n = 3,9054)	5.93 (SD 1.56) hours/night
Year 2 (n = 3,414)	6.26 (SD 1.43) hours/night
Year 3 (n = 3,067)	6.43 (SD 1.40) hours/night

PAP, positive airway pressure.

^*^Adoption is defined as the percentage of patients in Year 1 who start PAP therapy out of those diagnosed as positive for sleep apnea during testing.

^**^90-day adherence is defined using the CMS criteria of the % of patients adopting therapy in the first 90 days of treatment using PAP therapy ≥70% of days for at least 4 hours/day within a consecutive 30-day period.

^†^1-year adherence is defined as the % of patients using PAP one year after adoption out of the patients who initially started using PAP.

^‡^2-year persistence is defined as the % of patients using PAP two years after adoption out of the patients who initially started PAP.

^§^Year 1 usage is evaluated between PAP adoption, defined as Day 0, and Day 365. Year 2 usage is evaluated between Day 366 to Day 730. Year 3 usage is evaluated between Day 731 to 1095.

During year 1, patients (*n* = 3,905; Table 4) used PAP devices an average of 5.49 days per week, with mean usage of 5.9 h per night. During year 2, patients (*n* = 3,414) used PAP devices an average of 5.8 days per week, with mean usage of 6.3 h per night. In year 3, patients (*n* = 3,067) used PAP devices an average of 6.0 days per week, with mean usage of 6.4 h per night. In terms of effectiveness of PAP therapy, the mean REI/AHI at baseline was 38.33 breathing events per h; within 90 days of adoption of PAP, this was reduced to 1.82 events/h (normal based on the PAP machine-calculated rAHI values) and remained low through 1-, 2-, and 3-years following treatment adoption, with mean values all below residual estimated AHI of 2 events/h (Table 5).

**Table 5:** Mean REI/AHI at Baseline and PAP machine-calculated residual estimated AHI (rAHI) values after treatment initiation for those who adopt therapy.

**Sample for each timepoint**	**REI/AHI/rAHI (events/hour)**
Baseline for those who do not initiate treatment (*n* = 773)	18.86 (17.36)
Baseline for those who initiate treatment (*n* = 4,134)	38.33 (25.82)
90 days post treatment initiation (*n* = 4,075)	1.82 (2.17)
1 year post treatment initiation (*n* = 3,904)	1.54 (2.53)
2 years post treatment initiation (*n* = 3,405)	1.44 (1.91)
3 years post treatment initiation (*n* = 3,090)	1.41 (3.27)

AHI, Apnea-Hypopnea Index; REI, Respiratory Event Index; rAHI, PAP machine-calculated residual estimated Apnea-Hypopnea Index.

## Discussion

In this real-world analysis, quality of OSA care was high, as evidenced by shorter length of time between treatment steps, including time to diagnosis and time to treatment initiation after diagnosis. Length of time between steps was shorter than published reports citing long wait times and significant barriers in patients getting tested and on PAP treatment. Further, rates of both short- and long-term adherence and persistence to PAP therapy (up to 3 years) were also higher than what has been found in most prior studies, suggesting that a comprehensive, telehealth OSA care approach can effectively help more patients initiate therapy and then support them to remain in therapy and maintain long-term adherence. Importantly, PAP usage also improved significantly across 3 years, increasing the days per week and time per night that patients used their devices. Given that American Academy of Sleep Medicine clinical guidelines are clear that OSA is a chronic disease requiring life-long care, evaluating long-term outcomes is critical (Patil et al., 2019).

In recently published data, a higher proportion of patients receiving care in the comprehensive OSA care model described in this paper were satisfied with all measured points in the patients' journey, including ease of navigating the testing process, time between diagnosis and CPAP adoption, and availability and level of ongoing CPAP support, as compared to more traditional OSA care models (Salinas et al., 2025). These data, in conjunction with the real-world data presented here, highlight the importance of a comprehensive, clinically-integrated OSA care model for key stakeholders.

Given this was a real-world sample without a control arm, it is important to consider present results in the context of past literature. For example, in terms of the patient journey, many sleep centers have lengthy delays (Flemons et al., 2004). By contrast, patients in this real-world care program underwent telehealth consultation with a board-certified sleep medicine specialist within 5 days of completing a screening questionnaire and subsequently received HSAT results 12 days later. Patients typically initiated PAP within 8 days of receiving a diagnosis and treatment recommendation to begin PAP therapy. This is a more expeditious patient journey than has previously been reported. For example, a 2022 analysis related to the OSA diagnosis and treatment interview process suggested a timeframe of 1–2 months between each step on the OSA patient journey, with the investigators citing disorganization and a lack of communication between healthcare providers as reasons for delays in care (Ye et al., 2022). In a different review of data from five countries (Flemons et al., 2004), the US average wait time from the step of patient referral to sleep testing ranged from a few weeks to a year, not taking into consideration the time from testing to treatment adoption. Supplementary material to this article has been provided to observe differences in methodology and results for determining the average wait time.

Recent research on the clinical impact of reducing wait times for OSA care shows that more timely initiation of PAP therapy is associated with greater adherence to therapy. More specifically, earlier initiation of treatment has been associated with higher PAP adherence and greater improvements in daytime sleepiness and patient satisfaction (Pelletier-Fleury et al., 2004; Thornton et al., 2020). These researchers suggested that delayed care may change patient perceptions about the importance of sleep-disordered breathing, modify patient behavior (i.e., decrease use of PAP) and result in worse health outcomes.

In addition to speed, present results also demonstrate clinical quality, as most (80.6%) patients met CMS adherence criteria within 90 days, with 82.6% and 74.2% of patients remaining adherent 1 and 2 years later, respectively. These rates of adherence are meaningfully higher than those observed in traditional sleep care models (Risk Strategies Consulting, 2024; Weaver and Grunstein, 2008). Although PAP is the first-line recommended treatment for OSA due to its efficacy, its effectiveness is dependent on adherence, which historically has been disappointingly low, especially in long-term analyses (Weaver and Grunstein, 2008; Park et al., 2023; Cistulli et al., 2019; Kribbs et al., 1993).

The impact of nonadherence to treatment is far reaching. Data demonstrate that PAP adherence results in clinically significant improvements in multiple health outcomes. A 2019 systematic review found that successful treatment of OSA is associated with favorable economic outcomes (Wickwire et al., 2019). Nonadherent patients with sleep apnea also cost more than their adherent counterparts, resulting in higher total healthcare resource utilization and resulting higher total healthcare costs. Previously published data shows that adherence in the comprehensive care program associated with lower total health care resource utilization and healthcare costs reduced by $2,743/year on a risk-adjusted basis (Risk Strategies Consulting, 2024).

This study possesses numerous strengths. Most important, this is the first study to describe and provide key quality of care findings from a real-world, comprehensive, telemedicine sleep disorders management program that has been developed and refined over 10 years. Findings provide important data for multiple stakeholders, including employers who are a key stakeholder in the sleep disorders and chronic disease management ecosystem despite only recently investing in workplace sleep management programs. Other strengths include a large sample size of real-world data, long-term follow-up (3-years), and geographic diversity in the US.

At the same time, present results must be interpreted within the context of its limitations as real-world data also presents challenges including potential bias and confounding factors that differ from highly controlled randomized clinical trials. Most important, the observational study design precludes determination of causality. Data were captured as part of routine clinical care within an enterprise context; patients represent a non-randomized, consecutive sample of actual employees from workplaces across the United States, seeking evaluation and treatment of sleep disorders. While the analyzed patient population included a substantial number of employees working in both regulated and non-regulated industries, from a range of workplace environments with broad geographical distribution, generalizability of our findings is unknown. Analysis comparing patients from the regulated and non-regulated industries would be useful and informative, but is not feasible as it may violate our confidentiality agreements with the patients and their employers. Data from Study 1 was collected during the COVID pandemic (2021–2023), which we know was a time when healthcare utilization patterns varied; in many cases non-COVID healthcare utilization decreased but this care model provided care via telemedicine and may have been more protected from those downward trajectories; some research showed non-COVID related healthcare utilization drop but some telemedicine services increase (Cox et al., 2025). Further, bias may have been introduced by including patients for whom CPAP adherence is required for employment. This care model treated patients for whom adherence reporting was mandatory, as well as those who self-referred and did not have their adherence rates reported back to employers.

A final potential limitation may exist around AHI severity as this sample started treatment, on average, with a high REI/AHI. Although the field is somewhat mixed, with some findings indicating AHI severity may affect adherence (Park et al., 2023), and other findings suggesting that AHI severity did not predict adherence (Lykouras et al., 2024), it is worth noting that this sample did score as severe per baseline REI/AHI. This could potentially introduce bias, although it may also accurately represent that those seeking treatment are often in some level of distress and more symptomatic. As with all real-world studies, clinical and treatment variables reflect actual care delivery and clinical outcomes, without randomization and with a higher priority on minimized patient burden.

## Future directions

The results present important directions for future research. First, in terms of engagement, it is vital to identify patient-centered, provider/health system, and payor factors that support treatment adoption, as well long-term adherence. Patient-specific factors worthy of investigation include disease severity, presence of comorbid conditions, location, gender, and physiology. Personal characteristics including mental health, patient preferences, motivations, and socio-economic status may play a role in adherence. Non-patient factors are also important to consider, including aspects of employment or payor structure (e.g., industry, job role, plan design). Greater insights into each of these domains will help guide delivery of personalized care and improve outcomes for stakeholders. Related to this, greater insights into care delivery, including opportunities to leverage technology, advanced diagnostic technologies (e.g., multi-channel home sleep apnea testing with parameters that increase diagnostic sensitivity), artificial intelligence, and supportive care (e.g., sleep care navigators) will all help to streamline sleep care and optimize the patient journey. Finally, there is a great need for increased emphasis on outcomes that matter to patients, employers, and health systems more broadly, including impact on comorbid chronic diseases, improved workplace productivity (e.g., using employer-specific metrics), and reduced healthcare expenses, including healthcare resource utilization and costs. Development of such insights will require close partnership with multiple stakeholders including forward-thinking employers, health systems leaders, and payers. Such efforts are actively underway.

## Data Availability

The datasets presented in this article are not readily available because of confidentiality restrictions. Requests to access the datasets should be directed to fthorndike@noxhealth.com.
