# Supplementary material for: Managing sleep apnea: long-term outcomes from a comprehensive, patient-centered treatment care pathway
Source: Front Sleep. 2025 Jun 10;4:1593874. doi: 10.3389/frsle.2025.1593874 (PMC12713843; doi:10.3389/frsle.2025.1593874)
Supplement: Supplementary file 1 [file Supplementary_file_1.pdf]

Studies in the U.S., Canada, and Australia demonstrate variable wait times, but can range from 20 days to 12 months in between initial appointment and treatment

**Publication**	Riney et al., 2025	Flemons et al., 2004	Ye et al., 2022	Wickwire et al., 2024	Thornton et al., 2020	Rotenburg et al., 2010	Corrigan et al., 2022	Fatima et al., 2023	Fatima et al., 2025
Study location	USA	USA	USA	USA	Canada	Canada	Canada	Canada	Canada / Australia
Wait time	Days; median	Months; range	Months; estimate	Days; median	Days; mean	Months; mean	Days; mean	Weeks / months	Months
**Treatment Steps**									
Days elapsed from OSA screening to completion of teleconsultation with a physician	5 days	2-10 months (US only, table 1)	n/a	n/a	Time from referral to initial visit: 88 days<br><br>Time from referral to treatment: 123 days	11.6 months	Urban Residence:<br>Time to dx**: 20 days<br>Time to tx**: 62 days<br><br>Rural Residence:<br>Time to dx: 37 days<br>Time to tx: 85 days	Time taken to first see provider:<br><6 months 26%<br>6-12 months 15%<br>1-5 years 32%<br>>5 years 26%<br><br>Time from first assessment to diagnostic test:<br><2 weeks 34%<br>2 - 4 weeks 38%<br>1 - 3 months 19%<br>4 - 6 months 4%<br>>6 months 2%<br><br>Time from diagnosis to treatment:<br><2 weeks 30%<br>2 - 4 weeks 36%<br>1 - 3 months 27%<br>4 - 6 months 3%<br>>6 months 3%	**Canada**<br>Time to first see provider:<br>>6 months: 61%<br><br>Time from first assessment to diagnostic test:<br><3 months: 59%<br><br><1 month: 31%<br>1-3 months: 28%<br>3-6 months: 15%<br>6-12 months: 9%<br>>12 months: 6%<br><br>Time from diagnosis to treatment:<br><3 months: 70%<br><br><1 month: 45%<br>1-3 months: 25%<br>3-6 months: 11%<br>6-12 months: 5%<br>>12 months: 3%<br><br>**Australia:**<br>Time to first see provider:<br>>6 months: 37%<br><br>Time from first assessment to diagnostic test:<br><3 months: 76%<br><br><1 month: 44%<br>1-3 months: 32%<br>3-6 months: 10%<br>6-12 months: 2%<br>>12 months: 2%<br><br>Time from diagnosis to treatment:<br><3 months: 75%<br><br><1 month: 46%<br>1-3 months: 29%<br>3-6 months: 8%<br>6-12 months: 3%<br>>12 months: 1%
Days elapsed from telehealth consultation with physician to implementation of Home Sleep Apnea Testing	12 days		1-2 months*	n/a					
Days elapsed from HSAT completion to review of HSAT results, diagnosis, and treatment recommendations	9 days		1-2 months*	Varied by care pathway:<br><br>Split-night: 28 days<br>Home sleep apnea testing (HSAT): 36 days<br>Polysomnography (PSG): 37 days<br>PSG-Titration: 58 days<br>HSAT-Titration: 75 days					
Days elapsed from positive OSA diagnosis to PAP initiation	8 days		1-2 months*						

* based on one participant's qualitative interview response

** paper does not provide information on the starting point for the wait times

PAPER 1 - Riney HD, Thorndike FP, Agustsson JS, et al. Managing sleep apnea: long-term outcomes from a comprehensive, patient-centered treatment care pathway. Front Sleep. 2025;4. doi:10.3389/frsle.2025.1593874.

PAPER 2 - Flemons WW, Douglas NJ, Kuna ST, Rodenstein DO, Wheatley J. Access to diagnosis and treatment of patients with suspected sleep apnea. Am J Respir Crit Care Med. 2004;169(6):668-672. doi:10.1164/rccm.200308-1124PP

PAPER 3 - Ye L, Li W, Willis DG. Facilitators and barriers to getting obstructive sleep apnea diagnosed: perspectives from patients and their partners. J Clin Sleep Med. 2022;18(3):835-841. doi:10.5664/jcsm.9738

PAPER 4 - Wickwire EM, Zhang X, Munson SH, et al. The OSA patient journey: pathways for diagnosis and treatment among commercially insured individuals in the United States. J Clin Sleep Med. 2024;20(4):505-514. doi:10.5664/jcsm.10908

PAPER 5 - Thornton CS, Tsai WH, Santana MJ, et al. Effects of Wait Times on Treatment Adherence and Clinical Outcomes in Patients With Severe Sleep-Disordered Breathing: A Secondary Analysis of a Noninferiority Randomized Clinical Trial. JAMA Netw Open. 2020;3(4):e203088. Published 2020 Apr 1. doi:10.1001/jamanetworkopen.2020.3088

PAPER 6 - Rotenberg B, George C, Sullivan K, Wong E. Wait times for sleep apnea care in Ontario: a multidisciplinary assessment. Can Respir J. 2010;17(4):170-174. doi:10.1155/2010/420275

PAPER 7 - Corrigan J, Tsai WH, Ip-Buting A, et al. Treatment outcomes among rural and urban patients with obstructive sleep apnea: a prospective cohort study. J Clin Sleep Med. 2022;18(4):1013-1020. doi:10.5664/jcsm.9776

PAPER 8 - Fatima D, Tsai WH, Corrigan J, et al. Exploring patient-borne costs and wait times for obstructive sleep apnea (OSA) care among rural and urban adults. Canadian Journal of Respiratory Critical Care and Sleep Medicine. 2023;7(1):21-27. doi:https://doi.org/10.1080/24745332.2022.2156936

PAPER 9 - Fatima D, Sweetman A, Lovato N, et al. Access and models of obstructive sleep apnea care: a cross-national comparison of Canadian and Australian patient survey data. Journal of Clinical Sleep Medicine. 2025;21(3):467-477. doi:https://doi.org/10.5664/jcsm.11414
